# Constraints on signaling network logic reveal functional subgraphs on Multiple Myeloma OMIC data

**DOI:** 10.1186/s12918-018-0551-4

**Published:** 2018-03-21

**Authors:** Bertrand Miannay, Stéphane Minvielle, Florence Magrangeas, Carito Guziolowski

**Affiliations:** 1 LS2N, UMR 6004, École Centrale de Nantes, Nantes, France; 2 CRCINA, INSERM, CNRS, Université d’Angers, Université de Nantes, Nantes, France

**Keywords:** Answer set programming, Regulatory network modeling, Omic data integration

## Abstract

**Background:**

The integration of gene expression profiles (GEPs) and large-scale biological networks derived from pathways databases is a subject which is being widely explored. Existing methods are based on network distance measures among significantly measured species. Only a small number of them include the directionality and underlying logic existing in biological networks. In this study we approach the GEP-networks integration problem by considering the network logic, however our approach does not require a prior species selection according to their gene expression level.

**Results:**

We start by modeling the biological network representing its underlying logic using Logic Programming. This model points to reachable network discrete states that maximize a notion of *harmony* between the molecular species *active* or *inactive* possible states and the directionality of the pathways reactions according to their activator or inhibitor control role. Only then, we confront these network states with the GEP. From this confrontation independent graph components are derived, each of them related to a fixed and optimal assignment of active or inactive states. These components allow us to decompose a large-scale network into subgraphs and their molecular species state assignments have different degrees of similarity when compared to the same GEP.

We apply our method to study the set of possible states derived from a subgraph from the NCI-PID Pathway Interaction Database. This graph links Multiple Myeloma (MM) genes to known receptors for this blood cancer.

**Conclusion:**

We discover that the NCI-PID MM graph had 15 independent components, and when confronted to 611 MM GEPs, we find 1 component as being more specific to represent the difference between cancer and healthy profiles.

## Background

The exponential increase of biological data (genomic, transcriptomic, proteomic) [1] and of biological interaction knowledge in Pathway Databases allows modeling cellular regulatory mechanisms. Modeling biological mechanisms is done, most of the time, using boolean or ordinary differential equation representations. Those approaches have shown their efficiency in cellular phenomena study [2], disease research [3, 4], and bio-production optimization [5]. However, those modeling approaches cannot take into account the large amount of OMIC data. This limitation requires that the researcher preselects the OMIC data and network, adding bias to the analysis [6]. A classical way to perform OMIC data preselection is to use differentially expressed genes [7], this leads to select genes by imposing common fixed thresholds while their activation threshold may be specific for each gene. As a consequence the selected pathways may not be specific for the biological problematic. A common way to perform network preselection consists on choosing specific pathways according to the type of data and the biological problematic. Moreover, several regulatory databases such as KEGG, CBN, and Reactome [8–10] allow to select specific (e.g. apoptosis) pathways directly. Nevertheless, this network preselection approach can hide unsuspected pathways, reducing the possibility to discover new ones.

Some of the methods that identify subnetworks or network components, recognize specific pathways based on differentially expressed genes [11]. However, this kind of approaches considers pathways independently, and does not take into account the interactions between biological compounds. Other methods were developed to find involved pathways by identifying subgraphs or network clusters [12] from a regulatory network using topological informations and then use the gene expression profiles (GEPs) to identify a specific cluster. The majority of such methods uses protein-protein interaction (PPI) networks and GEPs to identify subgraphs [13, 14]. Those methods consider the interactions between biological compounds but infer protein states based on the associated GEP. That is, the built subgraph contains expressed proteins (obtained from associated genes expression) and their interactions [14]. These methods assume that a correleation between gene expression and protein activity exists, which is not necessarily true since an increase on gene expression can account of an increase of protein quantity, however in order to increase the activity of a protein another (e.g. phosphorylation) mechanism may need to be included. Methods using PPI networks are limited since they do not consider causality logic and different interaction roles. While the notion of causality is used by methods such as [15] to find a subgraph which maximizes the genes expression variation information; to our knowledge few subgraph identification methods based on GEPs consider direct interactions in regulatory networks, and much less include the different kind of interaction role (activation or inhibition) [16]. Moreover, the majority of those methods study protein interactions based on GEPs and without taking into account the difference between transcriptional and post-translational regulation. Finally, approaches that include the interaction role in their integrative analysis to link regulatory networks with GEPs [16, 17] use a local strategy, that is, they analyze sequentially each node in the graph with respect to its predecessors.

In this study we propose a method based on exhaustive and global graph coloring approaches [18]. These approaches are able to predict the graph coloring configurations, in terms of discrete states (e.g. active or inactive) of the molecular species of a biological network with respect to a set of experimental observations. In this work we extend those approaches by looking for *harmonious* or *perfect* colorations. The intuition behind the harmonious or perfectness notion is to point to reachable network discrete states that maximize the agreement between the molecular species *active* or *inactive* states and the directionality of the pathways reactions according to their activator or inhibitor control role. This can be expressed in natural language as follows: “for a given node in the graph we impose that its discrete active or inactive state is explained by a maximal number of regulators”. This statement is inspired from a hypothesis of redundancy in biological networks control, and we use Logic Programming to express this statement and search for coloring models where it holds for every node in the graph. Afterwards, we correlate the graph coloring models that maximize the perfectness notion and in this way build correlated graph *components*. After adding experimental data, our method is able to identify components of interest. We present an application of this method with transcriptomic data from myeloma cells (MC) of 602 MM patients and from normal plasma cells (NPC) of 9 healthy donors. Multiple myeloma is a hematologic malignancy representing 1% of all cancer [19] with a survival rate of 49.6% after 5 years. Our method of perfect graph colorings identification allowed us to identify 15 components. One of these components was statistically specific to MC in comparison to NPC. Using gene ontology enrichment analysis with the PANTHER tool we were able to associate this component to oncogenic phenomena.

## Methods

**Fig. 1:**
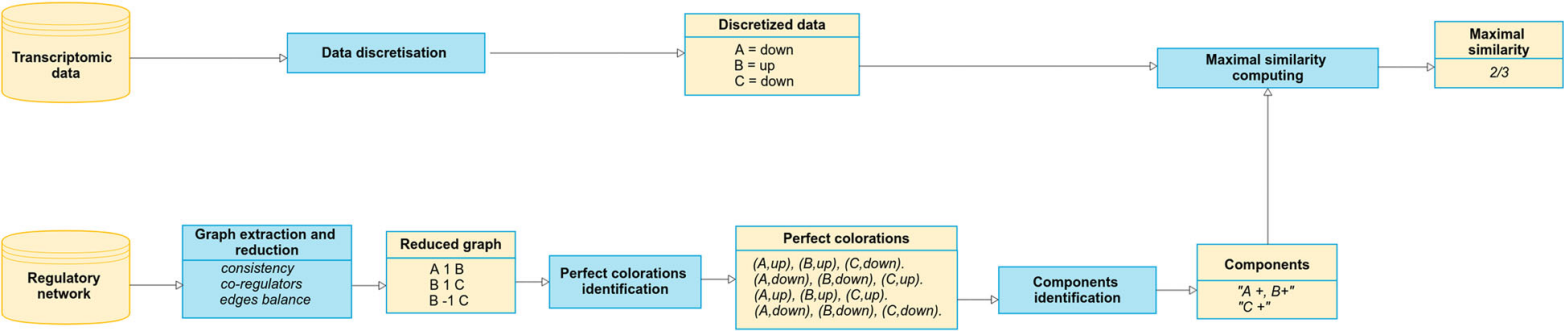
Overview of the perfect colorations modeling framework. Blue boxes refer to processing steps that are detailed in the Methods section and yellow boxes refer to input/ouput data

We propose in this paper a *perfect colorations* modeling framework which confronts a regulatory network with transcriptomic data (see Fig. 1). We detail in the following sections the main modeling steps of this framework. Note that the order of subsections does not follow the workflow due to the fact that some steps, in particularly the space solution reduction, require concepts which need to be introduced before. In Fig. 1 we illustrate the input (regulatory network and transcriptomic data) and output (maximal similarity and components) of our method. In “Toy example” section we present a toy example following step by step the workflow of Fig. 1.

### Answer Set Programming (ASP)

The perfect colorations identification is implemented in Answer Set Programming (ASP) [20]. This declarative programming approach allows us to express a problem in the form of a logic program (LP). The syntax of ASP is close to Prolog syntax because the grammatical structure of both LPs rules expresses a logical implication from the right terms of the rule towards the left terms of the rule. However, ASP semantics, which stands for the meaning of the vocabulary symbols used in each rule, allows a different type of solving mechanism. While in Prolog there is an inference process to search for an answer to a query, ASP programs allow to find all (Herbrand stable) models satisfying all the LP rules.

An ASP program consists of a set of predicates and first order logic rules of the form :



where Ai are atoms, i.e elements of the Herbrand base, which is composed of all the possible relations or predicates in first order logic of the LP. The Herbrand base is built by instantiating the LP predicates with the LP terms (constants or elements of the Herbrand universe). Basically, the line 1 explicits that A0 will be **trueif** A1,..., An are **true and** An+1,..., An+k **cannot be proven to be true** (not in the Herbrand base). In ASP, a solution or *answer set* is a *stable* Herbrand model, that is, a minimal set of true atoms without variables (grounded atoms) where all the logical rules are satisfied. We give now a brief description of the ASP rules used in this study; for deeper ASP understanding, please refer to [20, 21]. Variables in ASP start with uppercase letter whereas variables starting with lowercase letters denote constants. We use the following rule to generate candidate solutions:



This rule is satisfied when *n* predicates a(X,Z) are true, where X ranges over the domain of true predicates b(X) and Z is fixed by predicate c(Z). Another rule we use is expressed as:



This rule generates a predicate sum(X) where X is the number of predicates a(Z) which are true and ranged by the domain of true predicates b(Z). Finally, we used the following rule for optimization:



This rule expresses the selection of the answer sets with the minimal value of X, where predicate sum(X) is true. The “@*p*” indicates the optimization priority. The higher the value of *p*, the higher the priority.

### Modeling perfect coloring with ASP

#### Instantiation

**Graph:** a graph *G*(*V*,*E*) is composed of a set of nodes *V* and edges *E*. **Edge:** an edge is a tuple with 2 nodes (source and target), a sign (1 for activation, -1 for inhibition) and a weight.



**Node:** nodes are identified by the union of all sources and targets in the edges.



**Target:** a target is a node with at least one predecessor. We can identify those targets by looking for the union of all targets in the edges (line 12)



#### Candidate solutions generation

A colored graph is a graph in which all nodes are associated to a sign: up standing for “+” and down for “-”. These signs refer to the qualitative variation that one may experimentally measure in a molecular species (component of the graph) when comparing 2 cellular states, for example after v.s. before a stress condition. In this work we are interested on modeling sets of possible state variations of the components of the graph (line 16).



#### Definitions

##### Local consistent node coloring.

A node colored in a consistent way will be a node where its color is explained by at least one of its direct predecessor in the graph [18]. There are two possibilities for the coloring of a node *n* so that it will be explained by one of its predecessors *p*. This will depend on the sign of the edge from *p* to *n*. If the edge is an activation (line 17), *p* has to be associated with the same sign, otherwise if it is an inhibition (line 18), *p* has to be associated with the opposite sign. Because a node needs a predecessor to have a consistent color, this rule is only relevant for graph targets.



##### Imperfect target coloring.

An imperfect node coloring happens when a node is colored with a sign not explained by at least one of its direct predecessors in the graph.



##### Imperfect weighted regulator.

An imperfect weighted regulator *p* is a direct predecessor of a node *n* that does not explain consistently the color of *n*. The weight of this rule will be the weight of the edge from *p* to *n*.



#### Optimization constraints

Our method identifies graph colorings which minimize conflicts between target and predecessors, that is, it finds *perfect graph colorings* with minimal conflicts. In order to do this we apply 3 minimizations.

##### Inconsistency minimization

The first optimization will select the colored graphs with the minimal number of inconsistent targets. For this, we will first identify the inconsistent targets (line 23), then count the sum of those inconsistent targets (line 24). Finally, we will minimize this sum (line 25).



##### Imperfect target coloring minimization

The second optimization aims to reduce the solutions space to the graph with the minimal number of imperfect targets. In the same way as previously, the sum of imperfect target colorings is computed for each solution (line 26), then the solutions with the minimal number of imperfect colorations will be selected (line 27).



##### Imperfect weighted regulator minimization

The last optimization will minimize the sum of imperfect weighted regulators. First, for each target we compute the sum of the weights from the imperfect weighted regulators (line 28). Then we can compute the sum of weights for a colored graph (line 30). Finally, we can select the colored graph with the minimal sum of the weights associated to imperfect regulators (line 31).



### Component identification

Graphs or networks built from pathway databases, such as NCI-PID [22] are composed of nodes that can represent proteins, complexes, genes, transcription or proteins modification events. A *component* is defined as a set of molecular-species nodes which are color-dependent or color-correlated. That is, by fixing the color of one molecular-species node in this component, the colors of the other molecular-species nodes can be established so that the perfect coloring constraints hold. Given a graph, it is possible to identify its entire set of components by building a correlation matrix from the perfect coloring models obtained in “Modeling perfect coloring with ASP” section for each couple of nodes. Given a couple of nodes, 3 types of correlations are possible (Table 1). Positive correlation, *b*=0; negative correlation, *a*=0; and independent correlation *ab*≠0. Two nodes which are positively or negatively correlated will be incorporated in the same component.

**Table 1:** Correlation matrix informing about the dependence between two nodes colorations among perfect colorations. *a* and *b* inform for each coloring combination occurrence

Coloring	up	down
up	*a*	*b*
down	*b*	*a*

### Maximal similarity

This step computes the similarity between the components’ coloring and the dataset with the experimental observations present in one expression profile. Due to the perfect coloring framework and the fact that our model is based on a two-signs coloring, the nodes of a component *C*_*i*_ will have exactly two coloring configurations, we denote them by $C_{i}^{1}$ and $C_{i}^{2}$. $C_{i}^{1}$ will be the exact reverse of $C_{i}^{2}$ (the reverse of up is down and vice versa). We represent a dataset of experimental observations by a set of nodes in the graph with a fixed coloration obtained via a prior discretization of the experimental measurements. The maximal similarity (MS) computes the maximum, with respect to the size, of the intersection between the dataset of observations and each coloring configuration divided by the number of nodes observed in the component:

$${MS}_{i} = \frac{max\left(|{obs}_{i} \cap C_{i}^{1}|,|{obs}_{i} \cap C_{i}^{2}|\right) }{|{obs}_{i}|} $$

where *i* stands for the analyzed component and *obs*_*i*_ the experimental observations of nodes in the component *C*_*i*_.

### Space solution reduction

Due to our candidate solution generation, the space of solutions for a graph of *n* nodes will have a size of 2^*n*^. Because our graph coloring method is based on 2 signs with symmetric rules, we can observe that a coloring model and its reverse represents the same coloring perfectness. Therefore, it is possible to instantiate a node with a fixed color to reduce to half the solution space size. For example with line 32, we fixed the node node0 in the graph to down.



**Fig. 2:**
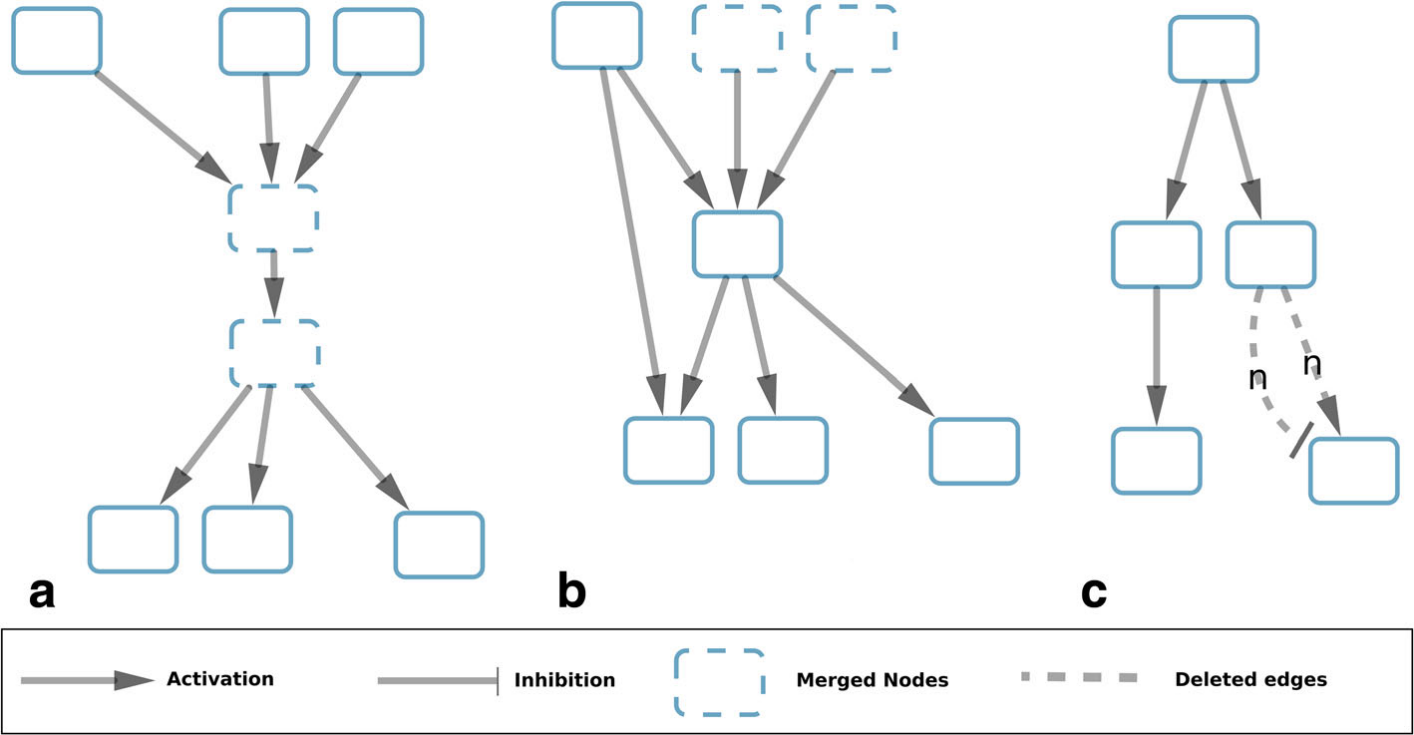
Patterns searched by the 3 reduction methods used in this study. **a**: nodes with correlated colors present in consistent solutions. **b**: nodes with correlated colors that share the same target. **c**: edges with the same weight, source, target and opposite signs

To furthermore reduce the complexity of the candidate solution space, we propose 3 graph reduction methods (Fig. 2) which can be applied successively over the graph prior to the perfect coloring ASP solving. These methods identify molecular-species nodes that will be in the same component, these nodes will be merged in a *subcomponent-node*. Subcomponents are derived through the topological reductions applied. Molecular-species nodes that belong to a subcomponent will be correlated to each other, and can also be correlated to molecular-species nodes belonging to other subcomponents. Therefore, a component, such as defined in “Component identification” section, can be composed by different (topological) subcomponents.

The first and second reduction methods identify subcomponents. Aggregating molecular-species nodes within subcomponent nodes reduces the number of nodes in the graph. The third method reduces the number of edges and detects components which are isolated of the rest of the graph.

#### Reduction based on the consistency (Fig. 2a)

This reduction method first identifies nodes which are candidates to have a sign correlation in consistent solutions, then it merges those nodes into a subcomponent-node. For that purpose we look for a specific pattern: a node with only one predecessor and a single incoming edge. This pattern will be merged into a component that will be composed of both elements and the sign of their correlation in a consistent solution (“+” if positive correlation, “-” for negative correlation). This process of pattern identification and merging of nodes into a subcomponent will be repeated until no new pattern is detected. Notice that the assembling of a subcomponent-node with a new molecular-species or subcomponent node generates a new subcomponent-node.

#### Reduction based on the co-regulators (Fig. 2b)

The second reduction identifies nodes candidates to have a sign correlation in candidate coloring solutions with minimized imperfect coloring. For this, we look for another pattern: two nodes without predecessors which share the same and unique successor (Fig. 2b). Those nodes can be merged into a subcomponent-node. In the same way as previously, the process of pattern recognition and then merging of nodes into a subcomponent will be repeated until no new pattern is detected.

#### Reduction based on the edges balance (Fig. 2c)

From both previous reduction methods we obtain a new graph composed of subcomponents. We consider here a non-merged molecular-species node as a subcomponent composed of one node. Then, we compute the edges weight between nodes of the graph by adding the weight of all the edges of the same sign that go from the molecular-species nodes of the source subcomponent to the molecular-species nodes of the target subcomponent. By merging together the edges of the same sign between two subcomponents, we may obtain subcomponents sharing at most 2 edges, *e*_1_ and *e*_2_, which are opposite signed and weighted respectively *w*_1_ and *w*_2_. In this case, we will compute new weights: $w_{1}\prime = w_{1} - min(w_{1},w_{2})$ and $w_{2}\prime = w_{2} - min(w_{1},w_{2})$. In case a new weight is equal to zero (Fig. 2c), we can delete the associated edge. After this edge reduction we may obtain disconnected subcomponents that are isolated from the graph. These subcomponents are color-independent of the rest of the graph and constitute a component as defined in “Component identification” section However, our method stores the information that targets of these components will be always consistent since they receive positive and negative interactions coming from the component. Also, on these targets, the perfectness constraint will not be verified.

### Implementation

To identify perfect graph colorings we used Answer Set Programming (ASP), namely clingo 4.5.4. The graph extraction from PID and the reduction algorithms were implemented with python 2.7 using the package NetworkX [23]. The components identification from perfect graph colorings were implemented in R [24] and python 2.7. All the computation (graph extraction, perfect coloration identification, components identification and MS computing) were made on a standard machine.

## Toy example

**Fig. 3:**
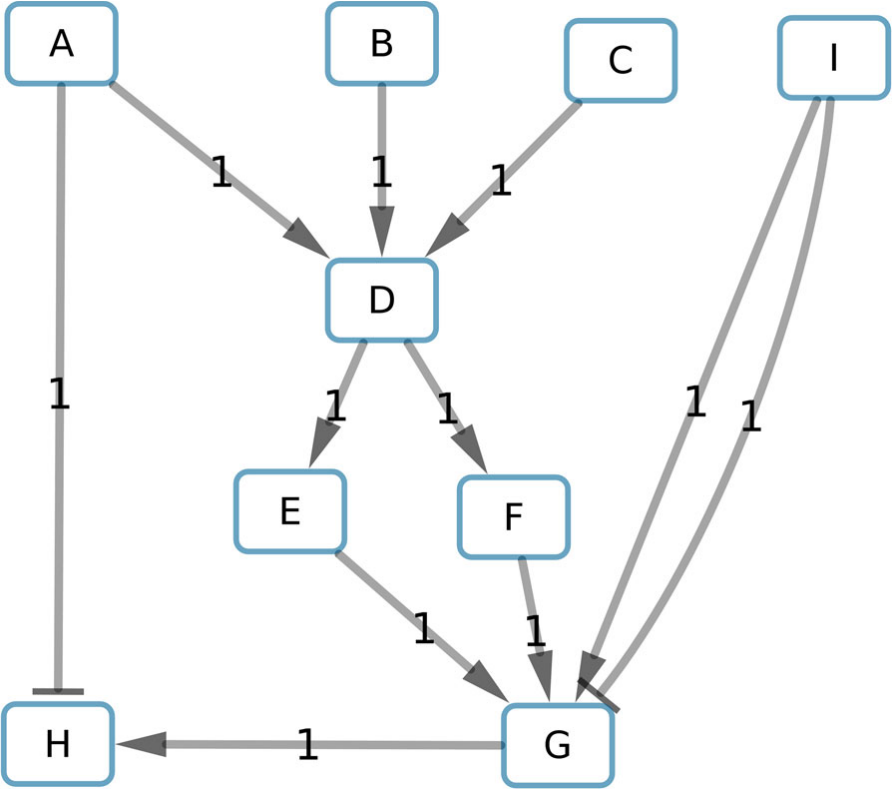
Toy example. Labels on the edges indicate the weight. Nodes are of molecular-species type; in this graph there are no subcomponent-nodes. Arrows head shaped as “ −>” (respectively “ −|”) mean activation (respectively inhibition)

To illustrate our method we propose a toy example with a graph composed of 9 molecular-species nodes and 11 edges (Fig. 3). To visually represent a subcomponent-node in our graphs we label it with the names of the molecular-species nodes it contains and their correlation signs in the subcomponent. For example, a subcomponent labeled “A +, B -” indicates that if A is associated to up (respectively down), B will be associated to down (respectively up).

### Graph reduction

**Fig. 4:**
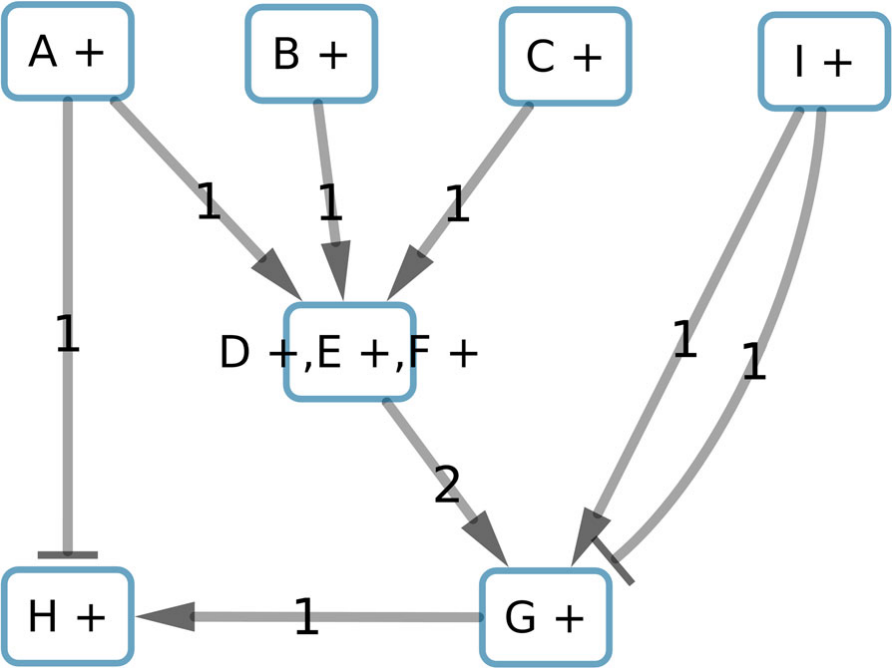
Result of the first reduction based on the consistency applied to graph in Fig. 3. All nodes of this graph are subcomponent-nodes

To reduce the solution space, we start by fixing the color of “A” to “+”, creating in this way a graph composed of subcomponents with only one molecular-species node and their respective correlation. Then, we apply the 3 methods previously described to reduce the graph size. The reduction based on the consistency merges the nodes D, E and F (Fig. 4) due to the fact that E and F have the same sign as D in consistent solutions.

**Fig. 5:**
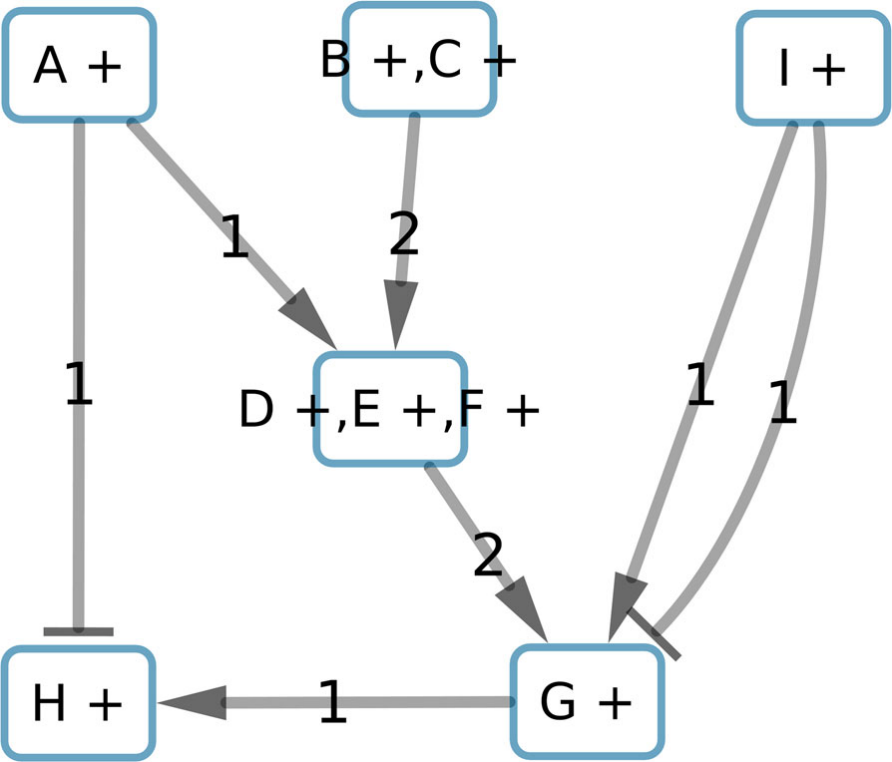
Result of the second reduction based on the co-regulators applied to the graph in Fig. 3

The second reduction, based on the co-regulators, identifies “B +” and “C +” as co-regulators of the component-node “D +, E +, F +”. Because these co-regulators do not have any predecessors and share the same unique successor, they can be merged into one component “B +, C +” (Fig. 5).

**Fig. 6:**
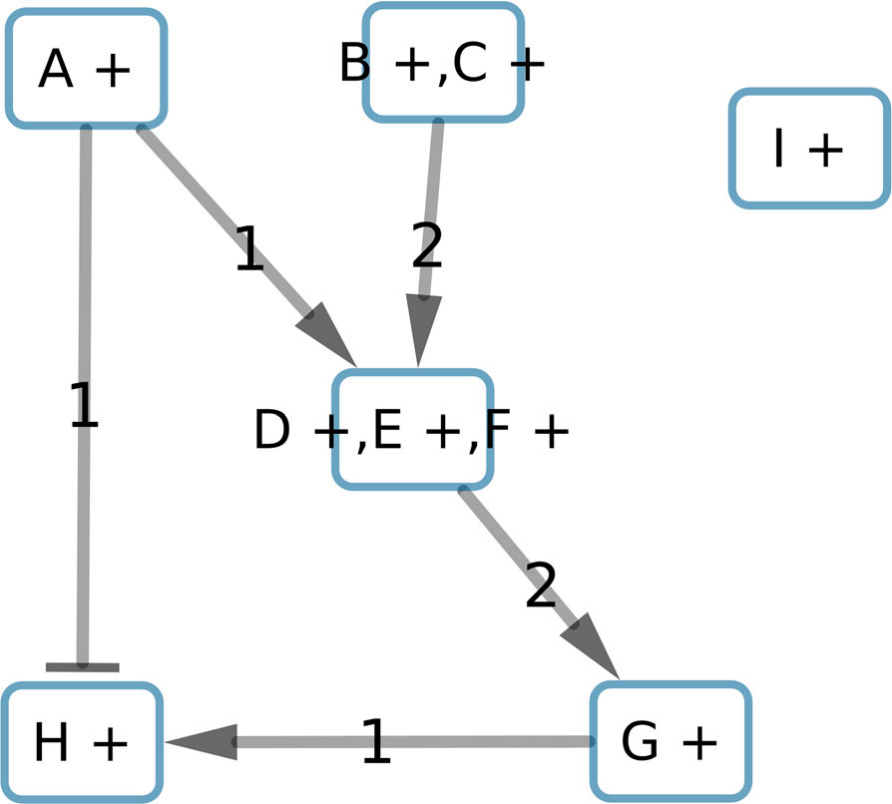
Result of the third reduction based on the balance of the weight of the edges

The last reduction, concerning balanced edge weights, identifies the edges from “I +” to “G +” which have the same weight and opposite sign. Those edges can be deleted, thus “I +” will be isolated of the rest of the graph and identified as a subcomponent independent of the rest of the graph. We consider “I +” as a component (Fig. 6). Moreover, we will store that “G +” will be consistent and imperfect independently of remaining predecessors due to the interactions with “I +”.

### Perfect coloring and components identification

With the reduced graph we can look for perfect colorings of the graph minimizing inconsistent patterns, then identifying the imperfect nodes colorations, and finally the imperfect weighted regulators. The results in Table 2 show the 2 perfect colorations for this example. With the instantiation of “A +” colored “down”, our method only proposes the coloration 1. However, we can notice that the coloration 2 is the reverse of coloration 1.

**Table 2:** Perfect colorations for the toy example graph and the space solution reduction

	A +	B +, C +	D +, E +, F +	G +	H +
Coloration 1	down	down	down	up	up
Coloration 2	up	up	up	down	down

**Fig. 7:**
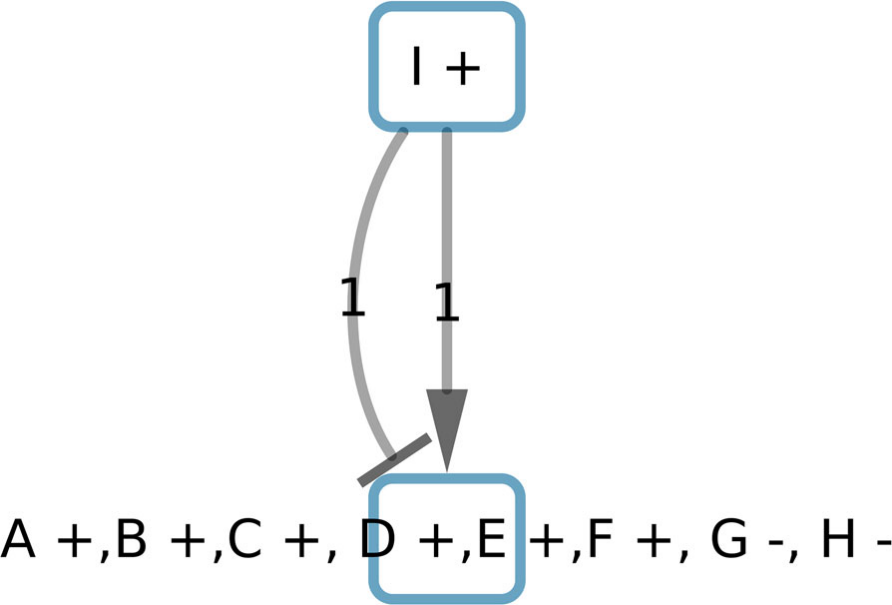
Result of components identification and their interactions. Edges represent constraints among the system variables to satisfy the perfect coloring constraints

We observe the subcomponents “A +”, “B +, C +” and “D +, E +, F +” have always the same coloration. Thus, we can merge those subcomponents. In the same way “H +” and “G +” have always the opposite coloration. They can be merged to a final component. This step will be done using matrix correlation methods for larger sets of nodes colorings. Finally, we identify the 2 components shown in Fig. 7.

### Maximal similarity computing

For a component, there are two possible colorings (component configurations) due to the symmetric property. For example, the component “A +,B +, C+, D +, E +, F +, G -, H -” (Fig. 7) has two possible configurations: *C*^1^={(*A*,*up*),(*B*,*up*),(*C*,*up*),(*D*,*up*),(*E*,*up*),(*F*,*up*),(*G*,*down*),(*H*,*down*)} and *C*^2^={(*A*,*down*),(*B*,*down*),(*C*,*down*),(*D*,*down*),(*E*,*down*),(*F*,*down*),(*G*,*up*),(*H*,*up*)}. Let us suppose a gene expression profile {*D*=*up*,*E*=*up*,*G*=*up*}. We can compute the similarity, *Sim*, between the expression profile and each coloring configuration as *Sim*_*C*_^1^=2 and *Sim*_*C*_^2^=1. The maximal similarity (MS) will be the maximal value between these two values divided by the number of observations in the profile, that is, *MS*=*max*(*Sim*_*C*_^1^,*Sim*_*C*_^2^)/3=2/3.

### Application

**Fig. 8:**
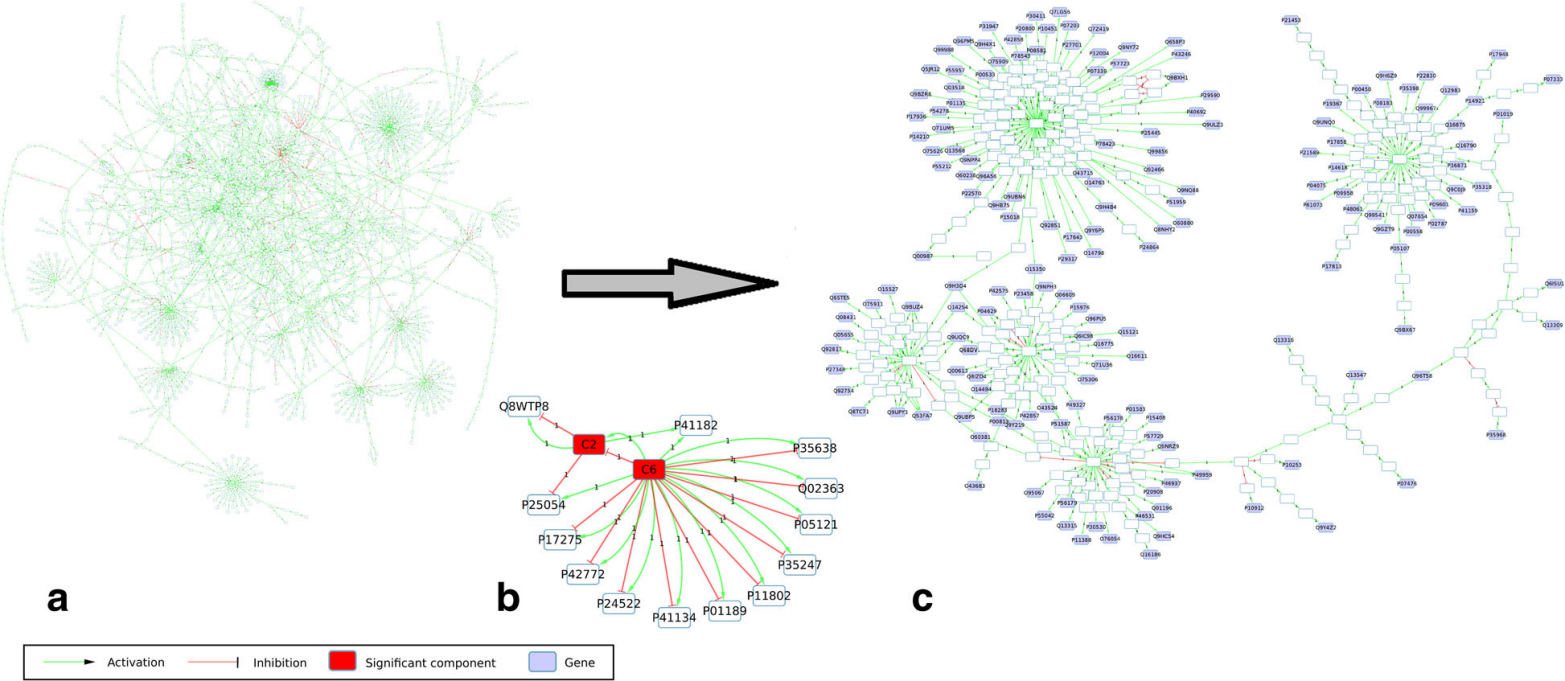
Overview of the application case. **a**: Subgraph obtained from the PID-NCI database, 2269 nodes and 2683 edges. CD40, IL6 and IGF1 are the 3 queried pathways connecting 529 differentially expressed genes. **b**: Components and their interactions. Simple components are labeled with the Uniprot identifiers of genes. The two most significant and larger components are colored in red. **c**: Graph representing the component 2, 422 nodes (167 genes) and 450 edges. Labeled nodes refer to Uniprot identifiers of genes. Unlabeled nodes refer to other biological compounds: proteins, complexes, transcription or proteins modification events

In this study we worked with gene expression profiles (GEP) issued from myeloma cells (MC) of 602 MM patients and from normal plasma cells (NPC) of 9 healthy donors used in a previous study [25]. For each GEP, we identified the over/under-expressed genes by comparison to NPC mean expression with a 1.2-fold. We choose this discretization threshold since it gives the best precision accuracy (lower than 2.2e-16) when making cross-validation tests with the MM GEPs (data not shown) using the sign-consistency approach described in [18]. Then, we use the PID-NCI database [22] to generate a graph by extracting the downstream events from three signaling pathways (IL6/IL6-R, IGF1/IGF1-R and CD40) [26] to the differentially expressed genes. The obtained subgraph from NCI-PID 2012, contained 2269 nodes, 2683 edges and connected 529 differentially expressed genes (Fig. 8a). The rest of the graph nodes were proteins, complexes, or proteins modification events.

## Results and discussions

### Perfect colorations

The graph reduction based on the consistency then co-regulators allowed to reduce the graph to 194 subcomponents and 408 edges. The edge weight computing and balance reduced the graph to 194 subcomponents and 389 edges. That is a reduction to 8% and 14% of the original number of nodes (2269) and edges (2683) respectively.

The perfect colorations method identified 16,384 coloring models (Table 3) for both graphs: the original and reduced. These models minimized inconsistency, imperfect nodes coloration, and imperfect weighted regulators. We can notice that the optimization results are the same for the initial and reduced graphs. However, the computation time of the original graph is larger than the reduced graph in 2 magnitude orders. In the perfect colorations identified by our modeling there were no inconsistent colorings. Only 1.5% of the targets of the original graph were imperfect (not explained by all predecessors). Finally, of the 35 imperfect targets, there was only one case where the number of imperfect regulators was of 2, the rest of 34 targets were found with only 1 imperfect regulator.

**Table 3:** Perfect coloration results for initial and reduced graph

Graph	# Nodes	#Targets	# Edges	Solution space	Number of inconsistent targets	Number of imperfect colorations	Number of imperfect weighted regulator	Computation time
Original	2269	2267	2683	2^2269^	0	35	36	4332 sec
Reduced	193	183	389	2^193^	0	35	36	14 sec

### Components identification

From those 16834 perfect colorations we identified 15 components (Fig. 8-b). 11 components were composed of 1 node (1 gene for each component), 2 were composed of 2 nodes (1 gene for each component), one was composed of 422 nodes (with 167 genes) and the last component was composed of 1832 nodes (with 349 genes).

### Components validation

Due to the fact that only two components are composed of more than one gene, we will focus mainly on those components (Table 4). For each gene expression profile *n* and each selected component *c*, we computed the maximal similarity: $MS^{n}_{c}$. Therefore, we obtained 611 vectors of 15 values.

**Table 4:** Results for the components analysis. The “Validation *p*-value” refers to the comparison between real and randomized data. The “Specificity *p*-value” refers to the comparison between MC and NPC data

Component	# Nodes	# Genes	Validation *p*-value	Specificity *p*-value
*C* ^2^	422	167	8,904e-03	0.019
*C* ^6^	1832	349	7.91e-05	0.573

In order to validate the similarity computing, we generated for each dataset, 5 randomized datasets by scrambling observed signs. As previously, for each randomized dataset, we computed the MS with the components configuration. Then, for each component, we compared the MS between real data and randomized data with a Welch’s t-test (Table 4, Validation *p*-value). Both components have a *p*-value lower than 0.05, allowing us to conclude to a statistical significance.

### Components specification

**Fig. 9:**
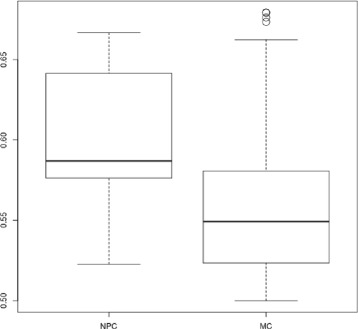
Boxplot of the MS computed with NPC and MM dataset for the component 2

**Fig. 10:**
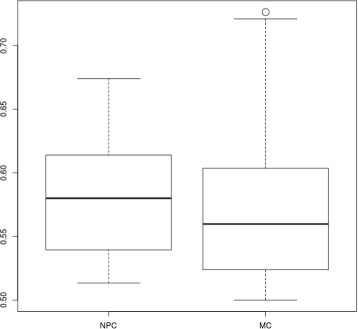
Boxplot of the MS computed with NPC and MM dataset for the component 6

The next step of the analysis was to identify specific component between MC and NPC. For this purpose, we compared the MS between the MC and NPC for each validated component with a Welch’s t-test (Table 4, Specificity *p*-value). *C*^2^ (Fig. 8c) was the only validated component with a *p*-value lower than 0.05. We can conclude that the MS for *C*^2^ is statistically different between MC and NPC (Fig. 9). For the component *C*^6^, the *p*-value was 0.5725747 (Fig. 10).

### Biological results

In order to link those analytic results to biology we used a Gene Ontology Enrichment Analysis [27] with the PANTHER Overrepresentation Test [28]. From a set of genes, this analysis can evaluate the biological processes over and under-represented in comparison to a random genes sample. We analyzed the genes set included in the components *C*^2^ and *C*^6^ (Tables 5 and 6).

**Table 5:** Five first results of the Gene Ontology Enrichment Analysis for the component *C*^2^

GO biological process	found	expected	Fold enrichment	*P*-value
regulation of cell death	75	11.98	6.26	6.46E-37
regulation of programmed cell death	73	11.21	6.51	8.33E-37
regulation of apoptotic process	72	11.11	6.48	4.90E-36
single-organism cellular process	149	77.70	1.92	9.90E-28
positive regulation of metabolic process	87	24.50	3.55	7.81E-26

**Table 6:** Five first results of the Gene Ontology Enrichment Analysis for the component *C*^6^

GO biological process	found	expected	Fold Enrichment	P-value
response to organic substance	182	42.74	4.26	1.02E-68
response to chemical	203	64.12	3.17	2.13E-57
response to oxygen-containing compound	129	23.26	5.55	1.32E-56
positive regulation of biological process	233	88.29	2.64	1.39E-55
regulation of cell proliferation	132	25.67	5.14	1.98E-54

The genes included in *C*^2^ (Table 5) seem strongly associated with cell death pathways: the three first biological processes are linked to cell death. Nonetheless, those pathways are strongly implicated in cancer disease [29]. On the other side, the component *C*^6^ (Table 6) does not look associated to redundant pathways since we cannot associate genes in the component *C*^6^ with a specific pathway. We notice however that *C*^6^ will describe cellular events linked with cell proliferation.

## Comparison with other clustering methods

In order to validate the components identification with our method, we compared it to two graph clustering algorithms: ClusterONE [12] and the Cytoscape plug-in ClusterMaker [30], which is a fuzzy c-means clustering algorithm. Both methods are similar to the one we propose since they do not include the GEPs for the clustering. We applied both methods with different parameters to the same regulatory network from the PID-NCI database used in our study. To estimate the quality of a clustering method, we consider a good cluster as one that is enriched with *specific* GO-terms based on the level of the GO-terms in the GO hierarchy. The higher the annotation level of the GO-term, the more specific its annotation will be. For each GO-term present in the ontology we computed its minimal depth from the root term (biological_process : GO:0008150); we found that the mean depth of all GO-terms was 7.07. We consider a GO-term to be *specific* when its minimal depth is higher than 7.07 Thus, for each cluster *i* we computed the Specific Enrichment (SE) index using the following formula:

$${SE}_{i}=\frac{|Specific Enriched {Terms}_{i}|}{|Enriched {Terms}_{i}|} $$

Where |*EnrichedTerms*_*i*_| is the sum of all enriched GO-terms (*P*.*val* ≤0.05) associated to the genes of the cluster *i* and |*SpecificEnrichedTerms*_*i*_| is the number of specific GO-terms enriched from the same list of genes. Based on this metric, we consider a good clustering method as one that produces larger and specific enriched clusters. Thus, we estimate for each clustering algorithm *c* the Clustering Quality (CQ) with the formula:

$${CQ}_{c}= \sum\limits_{i=1}^{n} {SE}_{i} * N_{i} $$

Where *N*_*i*_ stands for the number of genes in the cluster *i*. We compute the CQ with 5 clusterings: 2 obtained using ClusterONE (*CO*_1_ and *CO*_2_), 2 obtained using the fuzzy c-means algorithm (*FA*_1_ and *FA*_2_), and the last based on our component identification algorithm (CI). The parameters used to obtain the clusters and GO enrichment analysis were set as follows. For *CO*_1_ we used the basic parameters while we imposed to identify 2 clusters for *CO*_2_. For *FA*_1_ and *FA*_2_ we imposed the cluster search fixing 2 centers. In the case of *FA*_1_ we used overlapping genes for the GO enrichment analysis. We removed those overlapping genes for *FA*_2_. For our method, due to the fact that only the *components* 2 and 6 had more than one gene we consider the other *components* as outliers.

In Table 7 we show the results of this comparison. We observe that our clustering method seems more efficient to identify larger clusters enriched with more specific GO-terms (*CQ*=46.17). The specific enrichment score (SE) is shown low (< 0.08) in all the clusters obtained. This illustrates that only a low proportion of the GO-terms are specific in the PID-NCI graph. By comparison, when using no clustering method the SE of the full database is of 0.11. The *Loss information ratio* column in Table 7 shows a comparison with respect to the case where the full database was used to find specific terms. We compute this number for each clustering method *c* as 1−*CQ*_*c*_/*CQ*_*PID*_. This shows that our method is the one that obtains a higher proportion of quality score when compared to the full PID-NCI knowledge, and therefore a lower loss information ratio when compared to the 4 other clusterings. Finally, the closest clustering method (*FA*_1_) is based on overlapping genes which can lead to an overestimation of the *CQ* due to the fact that a gene associated with both clusters will be counted two times.

**Table 7:** Results of the comparison with other clustering methods

Clustering method	#clusters	#enriched clusters	#genes	*μ* ^*SE*^	*σ* ^*SE*^	*CQ*	Loss information ratio
*C*0_1_	105	24	344	0.10	0.155	37.82	35.8%
*C*0_2_	2	2	101	0.069	< 0.001	6.96	88.2%
*F* *A*_1_	2	2	688	0.065	0.02	44.94	23.8%
*F* *A*_2_	2	2	380	0.089	0.008	33.66	42.9%
*CI*	**15**	**2**	**511**	**0.089**	**0.006**	**46.17**	**21.6%**
PID-NCI graph	1	1	524	0.11	*∅*	58.93.	0%

The column #clusters stands for the sum of clusters. The column #enriched clusters stands for the sum of clusters which can be associated to enriched GO-terms. The column #genes stands for the number of genes in all the enriched clusters. The columns *μ*^*SE*^ and *σ*^*SE*^ stand for the mean and standard deviation for the SE value of the enriched clusters. The *Loss information ratio* column is computed for each clustering method *c* as 1−*CQ*_*c*_/*CQ*_*PID*_. The bold values refer to the results obtained with the component identification algorithm

## Conclusion

In this study, we proposed a method that imposes constraints to model graph coloration on biological signaling and regulatory networks. This method is able to reduce a regulatory network to subparts called components. These components describe network variables that are independent from others in the context of the *perfect coloring* constraints. Moreover, by using observations, we can select some of those components based on the maximal similarity between components configurations and those observations. The main points where our method is different from other subgraph identification methods are: (i) our method extracts network subcomponents by considering only the network logic (causality and inhibition/activation roles), while other methods consider topological features without logic; (2) the order of the analysis, our method first extracts logic network subcomponents states (harmonious colorings) and then confront these states to gene expression profiles (GEPs), adding less bias to the network v.s. data confrontation; and (3) when in a later step we integrate GEPs, we do it by locating GEPs measurements in the transcriptional layer, without overlapping transcriptional regulation with post-translational regulation. Using our method we were able to represent the species state variations (colorings) of a subgraph of the PID-NCI signaling and regulatory network (2269 nodes and 2683 edges) with 15 components. Each component will aggregate molecular-species having the same state-shift behavior given the PID-NCI graph topology. Only two (*C*^2^ and *C*^6^) of these 15 components include more than two molecular species nodes. From GO enrichment analyses, *C*^2^ is strongly associated to cell death pathways, this biological process is robustly associated to cancer. The *C*^6^ component cannot be associated to any specific pathway of cancer. Interestingly, this component specification was done independently of the GEP up-/down-regulation states. We have compared the identification of these 2 components by our method with respect to 4 other clustering results obtained with two different clustering methods on the same data. Our results show that our method retrieves larger and meaningful information, in the context of GO annotations associated to the genes within these components or clusters, than these other approaches.

When comparing the 611 gene expression profiles from myeloma cells, and healthy donors and shuffled data with the the genes present in the 15 components, we observed that *C*^2^ and *C*^6^ were the components which were significantly more specific to real data. Also, *C*^2^ was having a significant statistical specificity when compared unhealthy and healthy expression profiles.

Our method seems efficient to identify and select functional components specific to the gene expression profiles used in our study taking into account the computational complexity that represents analyzing large-scale networks. However in this case study the reduction to 15 components, with two validated ones with respect to shuffled data, does not allow us to provide a deeper understanding, especially with respect to the subtypes of patients based on the overall survival. As a perspective of this work, we wish to improve the subcomponent identification in order to be able to compute larger regulatory networks, and potentially full databases. For this purpose, we would like to implement the components identification in ASP. Another research line will be to apply this method to other data (regulatory network and observations data) as well as to model with other more refined modeling frameworks the subcomponent *C*^2^ to investigate the patient subtypes overall survival. One last perspective of this study could be to explore those targets wich are perfectly colored in all GEPs. This identification could be another strategy to improve the space solution reduction.

## References

[1] Marx V (2013). Biology: The big challenges of big data. Nature.

[2] Bentele M, Lavrik I, Ulrich M, Stößer S, Heermann DW, Kalthoff H, Krammer PH, Eils R (2004). Mathematical modeling reveals threshold mechanism in CD95-induced apoptosis. J Cell Biol.

[3] Liu W, Li C, Xu Y, Yang H, Yao Q, Han J, Shang D, Zhang C, Su F, Li X, Xiao Y, Zhang F, Dai M, Li X (2013). Topologically inferring risk-active pathways toward precise cancer classification by directed random walk. Bioinformatics (Oxford, England).

[4] Nevins JR (2001). The Rb/E2F pathway and cancer,. Human molecular genetics.

[5] Ates O (2015). Systems Biology of Microbial Exopolysaccharides Production. Front Bioeng Biotechnol.

[6] Mitra K, Carvunis A-R, Ramesh SK, Ideker T (2013). Integrative approaches for finding modular structure in biological networks. Nat Rev Genet.

[7] Dudoit S, Yang YH, Callow MJ, Speed TP (2002). Statistical methods for identifying differentially expressed genes in replicated cDNA microarray experiments. Statistica Sinica.

[8] Kanehisa M, Goto S (2000). KEGG: kyoto encyclopedia of genes and genomes. Nucleic Acids Res.

[9] Boué S, Talikka M, Westra JW, Hayes W, Di Fabio A, Park J, Schlage WK, Sewer A, Fields B, Ansari S, Martin F, Veljkovic E, Kenney R, Peitsch MC, Hoeng J (2015). Causal biological network database: a comprehensive platform of causal biological network models focused on the pulmonary and vascular systems. Database : the journal of biological databases and curation.

[10] Fabregat A, Sidiropoulos K, Viteri G, Forner O, Marin-Garcia P, Arnau V, D’Eustachio P, Stein L, Hermjakob H (2017). Reactome pathway analysis: a high-performance in-memory approach. BMC Bioinformatics.

[11] Haidari M, Zhang W, Wakame K, Papageorgiou LG, Vincent P, Fredlund E, Magnusson K, Nilsson H, Malyukova A, Rantala J, Klevebring D, Vinals F, Bhaskaran N, Zakaria S, Rahmanto A, Grotegut S, Nielsen M, Szigyarto C, Sun D, Lerner M, Navani S, Widschwendter M, Uhlen M, Jirstrom K, Ponten F, Wohlschlegel J, Grander D, Spruck C, Larsson L, Sangfelt O (2013). Disruption of endothelial adherens junction by invasive breast cancer cells is mediated by reactive oxygen species and is attenuated by AHCC. Life Sciences.

[12] Nepusz T, Yu H, Paccanaro A (2012). Detecting overlapping protein complexes in protein-protein interaction networks. Nature methods.

[13] Razi A, Afghah F, Singh S, Varadan V (2016). Network-Based Enriched Gene Subnetwork Identification: A Game-Theoretic Approach. Biomed Eng Comput Biol.

[14] Faisal FE, Milenkovic T (2014). Dynamic networks reveal key players in aging. Bioinformatics.

[15] Backes C, Rurainski A, Klau GW, Müller O, Stöckel D, Gerasch A, Küntzer J, Maisel D, Ludwig N, Hein M, Keller A, Burtscher H, Kaufmann M, Meese E, Lenhof HP (2012). An integer linear programming approach for finding deregulated subgraphs in regulatory networks. Nucleic Acids Research.

[16] Paull EO, Carlin DE, Niepel M, Sorger PK, Haussler D, Stuart J (2013). Discovering causal pathways linking genomic events to transcriptional states using Tied Diffusion Through Interacting Events (TieDIE). Bioinformatics.

[17] Nicolle R, Radvanyi F, Elati M (2015). CoRegNet: reconstruction and integrated analysis of co-regulatory networks. Bioinformatics.

[18] Thiele S, Cerone L, Saez-Rodriguez J, Siegel A, Guziołstrokowski C, Klamt S (2015). Extended notions of sign consistency to relate experimental data to signaling and regulatory network topologies. BMC Bioinformatics.

[19] Rajkumar SV (2016). Multiple myeloma: 2016 update on diagnosis, risk-stratification, and management. Am J Hematol.

[20] Lifschitz V (2008). What is answer set programming?. Proceedings of the 23rd National Conference on Artificial Intelligence - Volume 3.

[21] Gebser M, Kaminski R, Kaufmann B, Schaub T (2012). Answer Set Solving in Practice. Synthesis Lectures on Artificial Intelligence and Machine Learning.

[22] Schaefer CF, Anthony K, Krupa S, Buchoff J, Day M, Hannay T, Buetow KH (2009). PID: the Pathway Interaction Database. Nucleic acids research.

[23] Hagberg AA, Schult DA, Swart PJ. Exploring network structure, dynamics, and function using NetworkX. In: Proc 7th Python Sci Conf (SciPy2008). Pasadena: 2008. p. 11–15.

[24] R Core Team (2015). R: A Language and Environment for Statistical Computing.

[25] Miannay B, Minvielle S, Roux O, Drouin P, Avet-Loiseau H, Guérin-Charbonnel C, Gouraud W, Attal M, Facon T, Munshi NC, Moreau P, Campion L, Magrangeas F, Guziolowski C (2017). Logic programming reveals alteration of key transcription factors in multiple myeloma. Scientific Reports.

[26] Klein B (2010). Positioning NK-kappaB in multiple myeloma,. Blood.

[27] The Gene Ontology Consortium (2000). Gene Ontology: tool for the unification of biology. Nature Genetics.

[28] Mi H, Muruganujan A, Thomas PD (2013). PANTHER in 2013: modeling the evolution of gene function, and other gene attributes, in the context of phylogenetic trees. Nucleic Acids Res.

[29] Bold RJ, Termuhlen PM, McConkey DJ (1997). Apoptosis, cancer and cancer therapy. Surgical Oncology.

[30] Morris JH, Apeltsin L, Newman AM, Baumbach J, Wittkop T, Su G, Bader GD, Ferrin TE (2011). clusterMaker: a multi-algorithm clustering plugin for Cytoscape. BMC bioinformatics.

